# Colocalization of A_2a_ but not A_1_ adenosine receptors with GABA‐ergic neurons in cardiopulmonary chemoreflex network in the caudal nucleus of the solitary tract

**DOI:** 10.14814/phy2.13913

**Published:** 2018-11-22

**Authors:** Zeljka Minic, Donal S. O'Leary, Harry G. Goshgarian, Tadeusz J. Scislo

**Affiliations:** ^1^ Department of Physiology Wayne State University School of Medicine Detroit Michigan; ^2^ Department of Anatomy and Cell Biology Wayne State University School of Medicine Detroit Michigan; ^3^ Department of Emergency Medicine Wayne State University School of Medicine Detroit Michigan; ^4^ Cardiovascular Research Institute Wayne State University School of Medicine Detroit Michigan

**Keywords:** Adenosine receptors, cardiopulmonary chemoreflex, NTS

## Abstract

Adenosine operating in the nucleus of the solitary tract (NTS) may inhibit or facilitate neurotransmitter release from nerve terminals and directly inhibit or facilitate central neurons via A_1_ and A_2a_ pre‐ and postsynaptic receptors, respectively. However, adenosine A_2a_ receptors, may also activate GABA‐ergic neurons/terminals which in turn inhibit glutamatergic transmission in the NTS network. Our previous studies showed that adenosine operating via both A_1_ (inhibitor) and A_2a_ (activator) receptors powerfully inhibits the cardiopulmonary chemoreflex (CCR) at the level of the caudal NTS. A_1_ receptors most likely inhibit glutamate release in the CCR network, whereas A_2a_ receptors facilitate NTS GABA‐ergic mechanisms which in turn inhibit CCR glutamatergic transmission. Therefore, we hypothesized that A_2a_ receptors are located on NTS GABA‐ergic neurons/terminals whereas A_1_ receptors may be located on NTS glutamatergic neurons/terminals. We investigated this hypothesis using double immunofluorescent staining for A_2a_ or A_1_ adenosine receptors and GABA synthesizing enzyme, GAD67, in 30 *μ*m thick, floating, medullary rat sections. We found that A_2a_ adenosine receptors are localized within the GABA‐ergic cells in the caudal NTS, whereas A_1_ adenosine receptors are absent from these neurons. Instead, A_1_ receptors were located on non‐GABA‐ergic (likely glutamatergic) neurons/terminals in the caudal NTS. These data support our functional findings and the hypothesis that adenosine A_2a,_ but not A_1_ receptors are located on GABA‐ergic neurons.

## Introduction

Adenosine is a powerful modulator of cardiovascular reflexes including the arterial baroreflex and cardiopulmonary chemoreflex (CCR) which are primarily integrated in the nucleus of the solitary tract (NTS) (Mosqueda‐Garcia et al. 1989; Abdel‐Rahman and Tao 1996; Spyer and Thomas 2000; Scislo et al. 2001, 2008; Scislo and O'Leary 2005; Ichinose et al. 2009, 2012; Minic et al. 2014b). Adenosine is released into the NTS during life‐threatening situations such as ischemia, hypoxia, and severe hemorrhage (Winn et al. 1979; Van Wylen et al. 1986; Phillis et al. 1987; Yan et al. 1995; Scislo and O'Leary 2006; Minic et al. 2014a). During severe hemodynamic imbalance, intracellular adenosine accumulates as a result of adenosine triphosphate (ATP) catabolism within hypoxic neurons and glial cells. The important role of adenosine in modulating cardiovascular control at the level of the NTS is supported by the fact that the NTS contains the highest amount of adenosine uptake sites within the entire central nervous system (Bisserbe et al. 1985). The accumulated adenosine is released into the extracellular space and envelopes all neurons in the area (Winn et al. 1979; Van Wylen et al. 1986; Phillis et al. 1987; Yan et al. 1995; Scislo and O'Leary 2006; Minic et al. 2014a). Despite this global release, adenosine evokes specific and contrasting effects on regional sympathetic outputs and blood pressure control by acting on two antagonistic A_1_ and A_2a_ receptor subtypes which inhibit and facilitate neurotransmitter release, respectively, (Ralevic and Burnstock 1998; Scislo and O'Leary 2005). In the NTS, the facilitator A_2a_ adenosine receptors functionally prevail over inhibitor A_1_ adenosine receptors and this is evident by the observation that cardiovascular responses to exogenous adenosine (decreases in MAP and HR) are mimicked by agonists to A_2a_ receptors and not A_1_ receptor agonists which evoke pressor responses and sympathoactivation (Mosqueda‐Garcia et al. 1989; Barraco and Phillis 1991; Barraco et al. 1991; Abdel‐Rahman and Tao 1996). The specific actions of these two receptor subtypes is further highlighted by the observation that activation of A_1_ adenosine receptors within the NTS inhibits baroreflex control of hemodynamic and sympathetic responses while activation of A_2a_ adenosine receptors at the level of the NTS does not alter processing within the baroreflex arc (Scislo et al. 2008; Ichinose et al. 2009). The fact that global release of adenosine as well as microinjection of selective adenosine receptor agonists evokes such specific responses, supports the hypothesis that adenosine receptor subtypes may be preferentially located on specific neural networks within the NTS (Scislo et al. 2001, 2008; Scislo and O'Leary 2005; Ichinose et al. 2009).

The cardiopulmonary chemoreflex (CCR), also known as the von Bezold‐Jarisch reflex is triggered by the activation of mostly polymodal mechano‐ and chemosensitive C afferent fibers which transmit information from the cardiopulmonary area in the chest via vagus nerve to the NTS (Coleridge et al. 1973; Paintal 1973, 1977; Thorén 1977; Thorén et al. 1979a,b; Scislo et al. 1993). This reflex may be activated with serotonin (operating via 5HT_3_ receptors) which is released during cardiac ischemia from aggregating platelets forming coronary thromboses, or from ischemic endothelial cells (Oei et al. 1983; Burnstock et al. 1988; Evans et al. 1990). Other neuroactive substances, naturally released during cardiac ischemia, may also activate cardiac vagal afferents, for example: endogenous cannabinoids operating via TRPV1 receptors, ATP operating via P2x receptors, nicotine etc. (Evans et al. 1991; Wagner et al. 2001; Rocha et al. 2003; Lupinski et al. 2011). The CCR reactivity is enhanced during acute myocardial ischemia (Rocha et al. 2003; Lupinski et al. 2011).

*The CCR response*: vasodilation, bradycardia, and decreases in renal, adrenal and lumbar sympathetic nerve activities may be beneficial by decreasing afterload on the heart. However, profound activation of the reflex may also lead to severe hypotension and cerebral ischemia; this may lead to fainting and even to sudden cardiac death of young athletes (Mark 1983; Greenberg 1984; Ng and Maginot 2007). In the latter scenario, adenosine released into the NTS may act as a negative feedback regulator to fine tune this reflex and prevent the deadly over‐activation of the CCR.

In support of this model, our previous studies showed that adenosine operating via both A_1_ (inhibitor) and A_2a_ (activator) receptors powerfully inhibits the CCR at the level of the NTS. A_1_ receptors most likely inhibit glutamate release in the CCR network (Ichinose et al. 2012). Our recent studies indicated that adenosine A_2a_ receptors inhibit CCR transmission in the NTS (Minic et al. 2014b) and this inhibition occurs via a GABA‐ergic mechanism (Minic et al. 2015). A_2a_ adenosine mediated inhibition is markedly attenuated, that is, the CCR responses are restored following GABA‐ergic blockade in the NTS. This suggests that A_2a_ receptors may be located on the NTS GABA‐ergic neurons or terminals where they act to facilitate GABA release. Therefore, the present study tested the following hypotheses: (1) A_2a_ but not A_1_ receptors colocalize with NTS GABA‐ergic neurons/terminals; (2) A_1_ receptors are located on NTS non‐GABA‐ergic (most likely glutamatergic) neurons in the caudal NTS where the CCR is integrated. To test these anatomical hypotheses, we utilized the technique of immunohistochemistry to investigate the localization of adenosine A_1_ and A_2a_ receptors with respect to the NTS GABA‐ergic neurons/terminals expressing GAD67.

## Methods

All animal experiments were approved by the Institutional Animal Care and Use Committee of Wayne State University and were performed in accordance with the Guide for the Care and Use of Laboratory Animals from the Institute of Laboratory Animals Resources and National Institutes of Health guide for the care and use of Laboratory animals (NIH Publications No. 8023, revised 1978).

### Perfusion and tissue harvesting

Spraque‐Dawley rats (*n* = 3, Charles Rivers, Wilmington, MA) were anesthetized with intraperitoneal injection of urethane (Sigma, St. Louis, MO) and transcardially perfused using oxygenated Dulbecco's Modified Eagle's Medium/Ham F12 Medium (Sigma), followed by fixation in 4% formaldehyde (Fisher Scientific, Waltham, MA). The medulla oblongata was excised and postfixed for 24 h (4% formaldehyde). The tissue was then placed into 20% sucrose solution for 2 days or until the tissue sank to the bottom of the vial and was sectioned at 30 *μ*m using a cryostat (Leica Microsystems, Buffalo Grove, IL). The transverse sections were transferred to 0.1 mol/L phosphate‐buffered solution (Fisher) for immunohistochemical staining.

### Immunohistochemistry

All incubations and washes were performed at room temperature and on a shaker. The tissue sections were exposed to 10% normal horse serum diluted in immunobuffer (0.03% Triton in 0.1 mol/L phosphate buffer) to block unspecific antibody binding. Sections were then incubated in primary antibodies: a) mouse anti‐glutamic acid decarboxylase 67 (GAD67, 1:500, Millipore, Temecula, CA), b) rabbit anti‐A_1_ adenosine receptor (1:1000, Novus Biologicals, Littleton, CO), c) rabbit anti‐A_2a_ adenosine receptor protein (1:1000, GenWay Bio, San Diego, CA) for 24 h. The monoclonal GAD67 antibody was raised against the cytoplasmic form of the GAD enzyme. The experiments conducted by Millipore confirmed the selectivity toward GAD67 and no detectable cross‐reactivity with the GAD65 isoform of the enzyme (http://www.emdmillipore.com/US/en/product/Anti-GAD67-Antibody-clone-1G10.2,MM_NF-MAB5406). The polyclonal adenosine A_1_ receptor antibody was raised against a 20‐amino acid synthetic peptide corresponding to C terminus (https://www.novusbio.com/products/adenosine-a1-r-antibody-epr6179_nbp1-96749). The experiments conducted by Novus Biologicals showed detection of A_1_ adenosine receptor positive Purkinje neurons in rat brain sections. The monoclonal A_2a_ adenosine receptor antibody was raised against a peptide derived between 321 and 370 amino acids of the C‐terminus of adenosine receptor gene. The experiments conducted by the manufacturer in HepG2 cell lines showed a single band at approximately 46 kDa corresponding to the A_2a_ adenosine receptor protein (https://www.genwaybio.com/human-adora2a-antibody-85299). Sections were washed three times in tris‐PBS buffer and incubated overnight in secondary antibody: donkey anti‐mouse immunoglobulin conjugated to Alexa488 (1:200, Jackson) to visualize GAD67 reactivity. To visualize A_1_ or A_2a_ adenosine receptors, biotin‐conjugated donkey anti‐rabbit (1:500, Jackson) secondary antibody was incubated overnight followed by a 4‐hour incubation with Cy3‐conjugated streptavidin (1:1000 Jackson). Sections were mounted wet onto the microscope slides (Southern Biotech, Birmingham, AL) and the edges were sealed with clear nail polish.

### Image acquisition

Tissue sections were examined using a Zeiss Axioplan2 (Thornwood, NY) conventional microscope. The band widths for Alexa488 (green) and Cy3 (red) filters used on the microscope to visualize immunoreactivity were selected such to avoid bleed‐through. The excitation and emission spectrum for the green filter was set to: 490 and 525 ± 12 nm, respectively, while the excitation and emission spectrum for the red filter was set to: 595 and 620 ± 12 nm, respectively. Immunoreactive neurons were photographed using high‐resolution Zeiss AxioCam MRm digital camera controlled by Zeiss, Axiovision software v4.8. Image J software was used to adjust the brightness, sharpness, and contrast.

## Results

**Figure 1:**
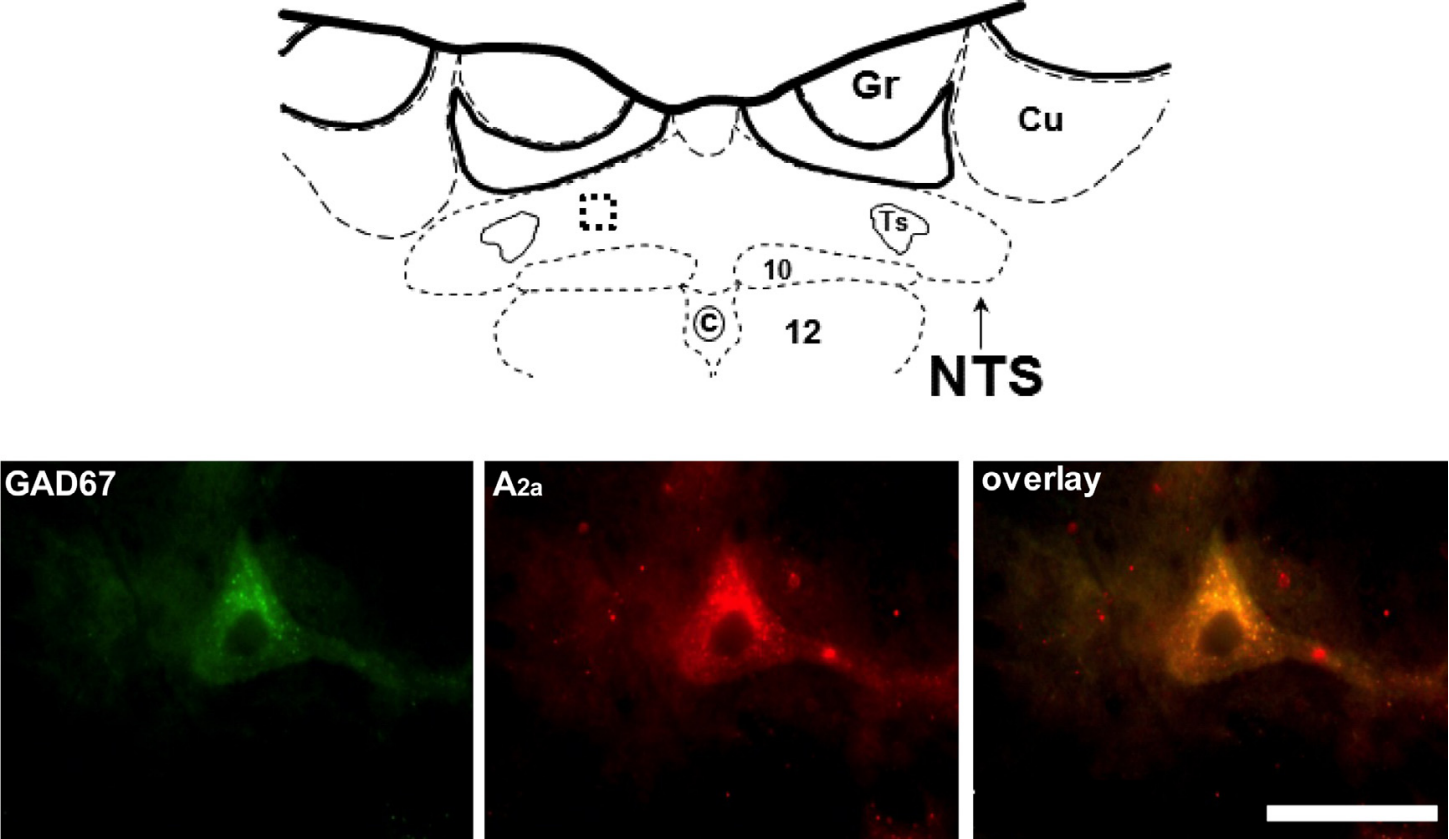
Immunofluorescent labeling for GAD67 (green) and A_2a_ adenosine receptors (red) in a 30‐*μ*m thick section of the caudal NTS. All three panels show the same field visualized with different filters: GAD67 is visualized using filter settings for Alexa488 and A_2a_ adenosine receptors are visualized using filter settings for Texas Red/Cy3 fluorophore. The area of the photomicrograph delineated by the box placed on the rat brain atlas schematic is shown in the enlarged view in the bottom panels. Gr, gracile nucleus, Cu, cuneate nucleus, Ts, tractus solitarius, 10, dorsal vagal nucleus, c, central canal, 12, hypoglossal nucleus. Left bottom panel depicts one neuron showing immunoreactivity for GAD67. Middle panel shows the same cell expressing immunoreactivity for A_2a_ adenosine receptors. Right panel shows overlay of the two channels and colocalization of A_2a_ adenosine receptor positive cell bodies with GAD67 synthesizing neurons. Scale bar = 50 *μ*m and applies to all the three panels.

**Figure 2:**
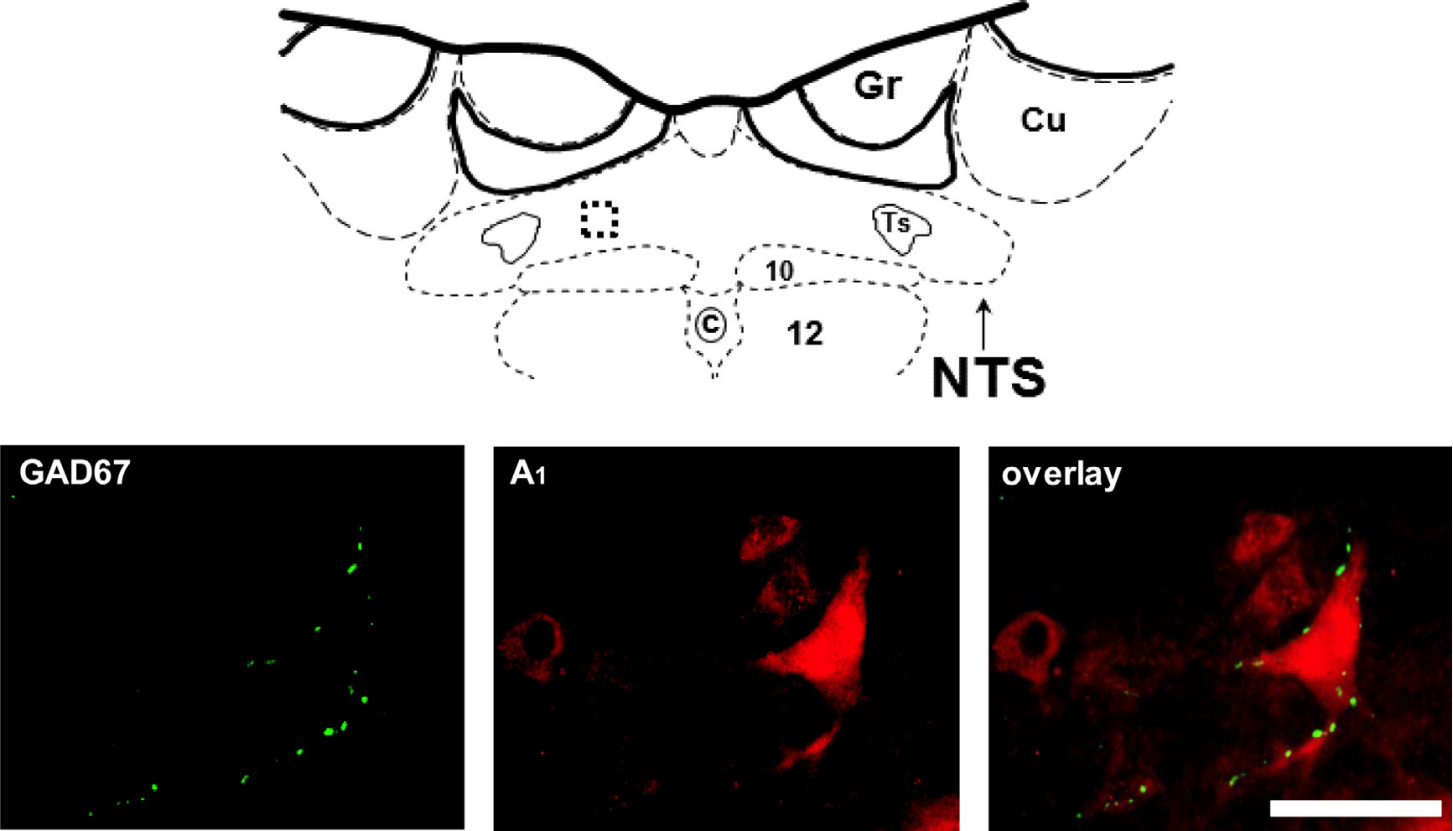
Immunofluorescent labeling for the GAD67 enzyme (green) and A_1_ adenosine receptors (red) in a 30 *μ*m thick sections of the caudal NTS. All three panels show the same field of view visualized using different filters: GAD67 is visualized using filter for Alexa488 (green) and A_1_ adenosine receptors are visualized using the filter for Texas Red/Cy3 fluorophore (red). The area of the photomicrograph delineated by the box placed on the rat brain atlas schematic is shown in the enlarged view in the bottom panels. Gr, gracile nucleus, Cu, cuneate nucleus, Ts, tractus solitarius, 10, dorsal vagal nucleus, c, central canal, 12, hypoglossal nucleus. Left bottom panel depicts GAD67‐positive axons/terminals. Middle panel shows five cell bodies showing immunoreactivity for A_1_ adenosine receptors. Right panel shows overlay of the two channels and absence of colocalization of A_1_ adenosine receptor positive cells with GAD67 neuronal terminals. Scale bar = 50 *μ*m and applies to all three panels.

The present study demonstrates distinct localization of adenosine A_2a_ and A_1_ receptor subtypes within the caudal NTS, where the CCR is integrated. A_2a_ adenosine receptors were expressed within GABA‐ergic neurons/terminals (Fig. 1), whereas A_1_ adenosine receptors were localized on non‐GABA‐ergic neurons some of which may receive GABA‐ergic input (Fig. 2).

GABA synthesizing neurons were identified by visualizing GAD67 immunofluorescence within the cell bodies or axon terminals in the caudal NTS. The top panel of Figure 1 is a schematic diagram of a brain section containing caudal NTS. The area of interest is delineated with the box. The bottom panels show a part of the box containing an NTS neuron expressing GAD67 (green, left) and A_2a_ adenosine receptors (red, middle) within the same field of view. The right panel shows overlay of the two channels and colocalization (yellow) of the GAD67 and A_2a_ adenosine receptors. We found positive A_2a_ adenosine receptor signal in 55 cells all of which also expressed GAD67.

Figure 2 represents a photomicrograph from a region of the caudal NTS delineated in the schematic and enlarged below. The bottom panels show GAD67‐positive axons/terminals (left panel), and A_1_ adenosine receptor positive cell bodies (middle panel) all within the same field of view. The right panel shows an overlay of the two channels. We found 64 A_1_ adenosine receptor positive cells, of which none expressed GAD67 suggesting that A_1_ adenosine receptors are not present on GABA‐ergic axons/terminals in the caudal region of the NTS. However, in some instances (as in Fig. 2), GABA containing axons were found in close proximity to A_1_ adenosine receptor positive cells.

## Discussion

The present study for the first time provided direct anatomical evidence for specific localization of A_1_ and A_2a_ adenosine receptors in the CCR network at the level of the caudal NTS. These data support our functional studies which suggest differential expression of A_1_ versus A_2a_ adenosine receptors on the NTS neurons involved in the processing of the CCR (Ichinose et al. 2012; Minic et al. 2015). The major finding of the study is colocalization of A_2a_, but not A_1_ adenosine receptors with GABA synthesizing neurons in the NTS. A_1_ adenosine receptors were expressed within NTS non‐GABA‐ergic neurons which receive GABA‐ergic projections. These observations were restricted to the caudal regions of the NTS involved in processing of the CCR information. These data further support the hypothesis based on our previous functional studies that adenosine A_1_ receptors inhibit CCR via direct inhibition of NTS neurons/interneurons mediating this reflex (Ichinose et al. 2012). In contrast, adenosine A_2a_ receptors inhibit CCR network via facilitation of neurotransmitter release from GABA‐ergic neurons in the NTS (Minic et al. 2015).

Adenosine receptor mediated GABA release at the level of the NTS and other CNS structures has been observed previously (Hettinger et al. 2001; Zaidi et al. 2006; Duy et al. 2010). Duy showed modulation of laryngeal chemoreflex by adenosinergic and GABA‐ergic mechanisms (Duy et al. 2010). Hettinger et al. (2001) demonstrated A_2a_ adenosine receptor modulation of GABA‐ergic signaling in striatum while Zaidi et al. (2006) reported A_2a_ adenosine receptor mRNA expression within GABA‐ergic neurons involved in respiration. A_1_ adenosine receptors, on the other hand, were found on NTS neurons surrounded by GABA‐ergic axons/terminals. Functional studies have demonstrated A_1_ adenosine receptor mediated inhibition of neurotransmitter release and inhibition of glutamatergic signaling, suggesting that NTS neurons on which A_1_ adenosine receptors were visualized in the present study may be of glutamatergic phenotype (Dunwiddie and Fredholm 1989; Banie and Nicholls 1993; De Mendonça et al. 1995; Scislo et al. 2008; Ichinose et al. 2012). These studies support our previous functional findings as well as the findings of the current study that both A_1_ and A_2a_ adenosine receptors are present in the caudal NTS and play an important role in differential modulating neurotransmitter release within the cardiovascular reflexes integrated there (Scislo et al. 2008; Ichinose et al. 2009, 2012; Minic et al. 2014b, 2015).

**Figure 3:**
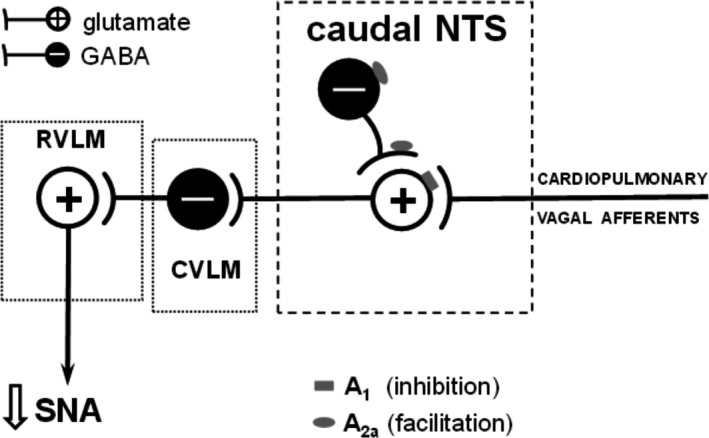
Location of A_1_ and A_2a_ adenosine receptor subtypes on the NTS neurons/terminals mediating changes in sympathetic nerve activity (SNA) characteristic for the cardiopulmonary chemoreflex. RVLM and CVLM are rostral and caudal ventrolateral medulla, respectively. A_1_ adenosine receptors (gray rectangles) may directly inhibit the CCR by acting on secondary NTS neurons within the CCR. Adenosine A_2a_ receptors (gray elipses) which are coexpressed on the GABA‐synthesizing neurons may inhibit the CCR by GABA release from the neurons and/or terminals linked to the CCR pathway in the NTS. Note that the CCR pathway is inhibited by both adenosine receptor subtypes although via different mechanisms.

In summary, the present neuroanatomical data as we predicted are based on previous functional studies and are summarized diagrammatically in Figure 3. A_1_ receptors are located on non‐GABA‐ergic (likely glutamatergic) neurons and directly inhibit the CCR network via inhibition on neurotransmitter release, whereas A_2a_ receptors indirectly inhibit neurotransmitssion in the CCR pathway via facilitating neurotransmitter release from GABA‐ergic neurons which then inhibit further neuronal activity in the CCR network.

Our data suggest, but do not prove, that A_1_ adenosine receptors inhibit the CCR via direct inhibitory action on NTS glutamatergic neurons mediating the CCR. Whether A_1_ adenosine receptors colocalize with NTS glutamatergic neurons which mediate the CCR should be addressed in future studies. The major limitation of this study is that the projection targets of GABA‐ergic and non‐GABA‐ergic neurons are not identified. GABA‐ergic neurons are most likely intrinsic NTS neurons involved in negative feedback present in the CCR and other cardiovascular reflexes integrated in the NTS (Zhang and Mifflin 2010). However, further studies should examine if NTS A_1_‐receptor‐expressing glutamatergic neurons may be labeled retrogradely from the caudal ventrolateral medulla, which mediates CCR. Since both A_1_ and A_2a_ receptors are present on vagal afferents terminating in the NTS (Castillo‐Melendez et al. 1994; Krstew et al. 1998) there is also possibility that A_1_ (inhibitor) but not A_2a_ (activator) receptors are located on vagal afferents mediating the CCR. If so, adenosine operating via A_1_ receptors could exert direct inhibition of glutamate release from CCR afferents as well as from NTS glutamatergic neurons (Castillo‐Melendez et al. 1994; Krstew et al. 1998; Ichinose et al. 2012). This possibility should be also addressed in future studies.

## Conclusion and Perspectives

The present neuroanatomical data support our previous functional findings suggesting that adenosine operating in the NTS via both A_1_ and A_2a_ receptors exerts powerful inhibition of the CCR. A_1_ receptors directly inhibit glutamatergic transmission in the CCR network whereas A_2a_ receptors exert indirect inhibition via facilitation of the release of GABA which in turn inhibits the CCR. The pronounced CCR‐induced bradycardia and depressor responses may lead to brainstem ischemia and release of adenosine as a product ATP breakdown within ischemic cells. Similarly, adenosine is released into the NTS during hypotensive stage of severe hemorrhage and alters hemodynamic and regional sympathetic responses to hemorrhage via both A_1_ and A_2a_ adenosine receptors (Minic et al. 2014a). In both situations, adenosine may prevent further dangerous decreases of arterial blood pressure and brainstem ischemia thereby aiding in the restoration of circulatory homeostasis.

## Conflict of Interest

Authors declare no conflicts of interest.
